# In vitro antioxidant and antidiabetic activity of essential oils encapsulated in gelatin‐pectin particles against sugar, lipid and protein oxidation and amylase and glucosidase activity

**DOI:** 10.1002/fsn3.1935

**Published:** 2020-10-07

**Authors:** Roghayeh Siahbalaei, Gholamreza Kavoosi, Raheleh Shakeri

**Affiliations:** ^1^ Institute of Biotechnology, School of Agriculture Shiraz University Shiraz Islamic Republic of Iran; ^2^ Department of Biological Sciences and Biotechnology Faculty of Sciences University of Kurdistan Sanandaj Iran

**Keywords:** essential oils, gelatin, glucose oxidation, lipid oxidation, pectin, protein oxidation

## Abstract

The in vitro antioxidant and antidiabetic activities of *Oliveria decumbens*, *Thymus kotschyanus*, *Trachyspermum ammi*, and *Zataria multiflora* essential oils incorporated into gelatin‐pectin composite were investigated. The gas chromatography–mass spectrometry characterization revealed that thymol (1.2%–86.4%), carvacrol (3.2%–52.4%), gamma‐terpinene (0.0%–12.7%), para‐cymene (3.2%–5.2%), geraniol (0.0%–14.5%), and spathulenol (0.0%–13.6%) are the major constituents of the essential oils. Gelatin‐pectin composite incorporated with the essential oils exhibited acidic pH (2.40–3.04), low conductivity (265–278 µS/cm), low surface tension (19.0–23.5 mN/m), low Newtonian viscosity (23.7–28.5 mPa.s), negative zeta‐potential (14.2–16.9 mV), and nanoscale particle size (313–336 nm). These rheological properties result in the production of globular gelatin‐pectin nanoparticles with a size range of 500–700 nm. The FTIR spectra of gelatin‐pectin and gelatin‐pectin‐essential oils to some extent were similar, suggesting the noncovalent interactions between them. Gelatin‐pectin composite incorporated with the essential oils displayed antiglucose oxidation (130–150 µg/ml) antilipid peroxidation (120–130 µg/ml), antiprotein oxidation (150–168 µg/ml), and antiprotein glycation (145–170 µg/ml) as well as antiamylase (216–230 µg/ml), and antiglucosidase (212–238 µg/ml) activity. The essential oils strongly improved the antioxidant capacity of the gelatin‐pectin composite so strongly which can be recommended as a natural compound for oxidative stress management.

## Graphical abstract

Oliveria, Thymus, Trachyspermum, and Zataria essential oils had antidiabetic activities. Essential oils displayed antilipid peroxidation and antiprotein oxidation activities. Essential oils displayed antiamylase and antiglucosidase activities. Gelatin‐pectin‐essential oil generates spherical and homogenous particle.

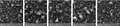

## 1 INTRODUCTION

Diabetes mellitus is mainly characterized by hyperglycemia, hyperlipidemia, and oxidative stress at sugar, lipid, and protein levels. Hyperglycemia can promote superoxide buildup through different metabolic pathways for instance; augmented glucose flux through the polyol pathway; increased protein glycation; protein kinase activation; hexosamine activation and decline in antioxidant defense system. Metabolic disturbance and oxidative stress which result from hyperglycemia have long been associated with neurological, urological, cardiovascular, kidney, and eye vision disorders (Chandra et al., 2019). Because of the rising number of diabetic patients and scarcity of the number of antidiabetic drugs without side effects, the efforts to find new drugs with no side effects have increased. The search for natural compounds with antidiabetic effects has increased around the world. In this regard, natural antioxidants can be used as an effective approach to manage diabetes as an oxidative stress‐related disease (Daneshzad et al., 2019).

Medicinal plants are excellent sources and producers of polyphenols, flavonoids, polyunsaturated fatty acids (omega‐3), functional amines, polypeptides, and other effective chemicals (Salehi et al., 2019). Among these phytochemicals, volatile oils are a mixture of lipophilic compounds, such as phenolic, terpenes, terpenoids, aliphatic alcohols, aldehyde, ketones, and flavonoids, all possessing numerous medicinal properties. The important properties of the volatile oils such as antimicrobials, antioxidants, and anti‐inflammatory have been proven (El‐Saber Batiha et al., 2020). The volatile oils are insoluble in water and are easily degraded by extreme acidity, light, and high temperature which limit their applications in the food and pharmaceutical industries (Ribeiro‐Santos et al., 2018). Encapsulation of volatile oils in natural proteins and carbohydrate polymers or in lipid droplets could decrease the volatility, increase solubility, and improve the stability and efficiency of these valuable phytocompounds (Bakry et al., 2016).

The plant *Oliveria decumbens Vent* is used to treat indigestion, diarrhea, abdominal pain, and infectious diseases. *O. decumbens* is an important source of antioxidant, anti‐inflammatory, anti‐cancer, and anti‐microbial materials and may be used for the treatment of infectious and skin diseases (Jamali et al., 2018). *Trachyspermum ammi* Linn is a highly valued medicinally important species. The fruits of *T. ammi* have been widely administered for liver, spleen as well as gastrointestinal disorders such as nausea, vomiting, reflux, and abdominal cramps. Modern pharmacological studies have shown that *T. ammi* possesses anti‐microbial, antioxidant, and anti‐inflammatory activities (Ranjbaran et al., 2019). *Thymus kotschyanus* Boiss and Hohen is widely used as a traditional treatment for digestive problems. It is used for the treatment of bronchitis, laryngitis, painful menstruation, colic, and hangovers. This plant has been externally applied to treat minor injuries (wound healing), mastitis, mouth, and throat and gum infections. Pharmacological studies have shown anti‐bacterial, hypotensive, and cardiotonic effects for this plant (Hosseinzadeh et al., 2015). *Zataria multiflora* Boiss is extensively used in Iranian traditional medicine for its various effects including as a carminative, stimulant, diaphoretic, diuretic, appetizer, pain killer, control of fever and treatment of dyspepsia, hysteria, sore throat, for the treatment of cough and whooping cough. Modern pharmacological studies have shown that this plant possesses a wide range of biological properties including antinociceptive, antimicrobial, anti‐inflammatory, and anticancer effects (Kavoosi and Rabiei, (2015). These plants are traditionally used to treat gastrointestinal distress and diabetes. These useful plants have valuable phytochemicals properties with broad traditional and medicinal uses including antimicrobial, antioxidant, anti‐inflammatory, and anticancer activities. But to the best of our knowledge, there is no scientific information about their antidiabetic properties.

Present study is focused on the chemical characterization of the volatile oils extracted from *O. decumbens* (ODEO), *T. ammi* (TAEO), *Z. multiflora* (ZMEO), and *T. kotschyanus* (TKEO). The essential oils were encapsulated in the gelation‐composite solution. The rheological properties of the gelatin‐pectin‐essential oils composite solutions were then studied. The gelatin‐pectin‐essential oil particles were prepared by electrospray and the morphology of the particles analyzed by scanning electron microscope. The in vitro antioxidant and anti‐diabetic capacities of the gelation‐pectin composite particles against glucose oxidation, lipid peroxidation, protein peroxidation, protein glycation, α‐amylase, and α‐glucosidase were analyzed.

## 2 MATERIALS AND METHODS

### 2.1 Plant materials, essential oils extraction and characterizations

The aerial parts of *O. decumbens* (herbarium number: 55,078) were collected from the mountainous areas of Fars, Iran (Jamali et al., 2020). *T. ammi* seeds were purchased from local perfumeries. The aerial parts of *Z. multiflora* (herbarium number: 24,985) were collected from the mountainous areas of Marvdasht town, Iran (Azadi et al., 2020). The aerial parts of *T. kotschyanus* (herbarium number: 65,110) were collected from mountainous areas of Fars Province, Iran. The identification of collected plants was thankfully done by Professor Ahmad Reza Khosravi, plant taxonomist at Biology Department, Shiraz University, Iran. The plant materials were dried in the shade for 3–4 days. The air‐dried plant samples were hydro‐distilled for 3 hr using Clevenger apparatus to collect the essential oils. Gas chromatography–mass spectrometry (GC‐MS) was carried out using Agilent gas chromatograph (Agilent 7890B GC 7955AMSD) coupled with a single quadrupole mass spectrometer and silica HP5MS column (30 m × 0.25 mm × 0.25 µm). The temperatures of the ion source and that of interface were 210°C and 270°C, respectively. The programmed temperatures of the oven were as follows: four min at 60°C, rising to 140°C at 20°C/min. Then, increased to 220°C at 10°C/min and finally fixed for 10 min at 220°C. Mass spectra were obtained at 80 eV with mass ranges of 50–400 m/z. The essential oil compositions were determined by comparing the fragmentation patterns of the peaks with libraries mass spectra (Wiley 7n and NISTO5A) (Oladimeji et al., 2016). The amounts of phenol content in samples were determined using gallic acid as standard. Essential oils were solubilized in DMSO at a concentration of 1,000 µg/ml gallic acid equivalent (Al‐Rubaye et al., 2017).

### 2.2 Preparation and characterization of gelatin‐pectin‐essential oil composite solution

A homogeneous solution containing gelatin (7 g), pectin (3 g), and 100 ml acetic acid (60%) was prepared and stirred continuously at 40°C. Gelatin‐pectin nanocomposite incorporated with plant essential oils was prepared by mixing the essential oil (100 mg/g of total polymer) with the gelatin‐pectin solution and stirred for 12 hr, at ambient temperature. Glycerol (100 mg/g of total polymer) and glutaraldehyde (10 mg/g of total polymer) were added to the composite solution then mixed thoroughly (Kavoosi et al., 2014). The conductivity and acidity of the composite solution were determined by the conductometer‐pH meter. Particles Zeta‐potential were calculated by Brookhaven Instrument (New York, USA) based on the phase analysis light scattering (PALS) method. The particles hydrodynamic diameters were calculated using a Brookhaven Instrument (USA) based on the principle of dynamic light scattering (DLS) method (Kavoosi et al., 2013). The surface tension of the composite solution was calculated by Du Nouy tensiometer (Kruss, Germany) at ambient temperature. The viscosities of the composite solutions were quantified using the MCR302 rheometer (Anton Paar) at a shear rate of 0.01 to 100 s^−1^, and the viscosity values were reported at 50 s^−1^.

### 2.3 Preparation of gelatin‐pectin‐essential oil composites particles

Electrospraying of the gelatin‐pectin solutions was carried out by full option electrospinning machine 5 (Full Option Lab2 ESII‐II, Nano Azma, Iran). Stainless steel needle was used as nozzle and a thin aluminum sheet used as the collector. The needle was connected to the positive polarity electrode of the high‐voltage power supply. The collector was attached to the grounding electrode. Gelatin‐pectin composite nanoparticles were fabricated by electrospraying diluted gelatin‐pectin solution under a fixed electrostatic field strength of 10 kV at a fixed length of 10 cm over a fixed collection time (10 min) with a feed rate of 0.3 ml/h. After coating with gold, the magnified images of the nanoparticles were recorded with a Tescan‐Vega3 scanning electron microscope (Tescan, Czech). Fourier transform infrared (FTIR) spectroscopy of nanoparticles was carried out using Bruker FTIR (Germany) in the range between 4,000 and 400 cm^−1^ (Homayouni et al., 2017). The dried particles were collected from aluminum sheet and dissolved (10 mg/ml) in dimethyl sulfoxide (DMSO, making 1.0% final concentration) for further studies.

### 2.4 Glucose autoxidation inhibition assay

Thirty microliters of different concentrations of essential oil particles (50, 100, 200, 300, and 400 µg/ml) were incubated with reaction solution containing sodium benzoate (1 mM), glucose (500 mM), and copper (II) sulfate (100 µM) at ambient temperature for 4 days. The fluorescence intensity of the solutions was read at 310 nm (excitation) and at 410 nm (emission). The percentage reduction in fluorescence intensity was taken as the percentage inhibition in glucose oxidation as follows: percent inhibition = [(fluorescence intensity in the absence of essential oil—fluorescence intensity in the presence of essential oil)]/fluorescence intensity in the absence of essential oil × 100. Ethylenediaminetetraacetic acid (EDTA, 1.0 mM) was used as a positive control (Hunt et al., 1994).

### 2.5 Lipid peroxidation inhibition assay

Thirty microliters of different concentrations of essential oils were incubated with 1,000 µl of LDL solution (1.0 mg/ml in phosphate‐buffered saline (PBS)). Instantly, 1,000 µl cupric sulfate (10 µM in PBS) as an oxidizing agent was added and kept at ambient temperature for 12h. The percent light absorbance reduction at 234 nm was taken as the percent of lipid peroxidation inhibition as follows: percent inhibition = [(Absorbance in the absence of essential oil − Absorbance in the presence of essential oil)]/Absorbance in the absence of essential oil × 100. Butylated hydroxytoluene (BHT, 10 mg/ml) was used as control (Amarowicz & Pegg, 2017).

### 2.6 Protein oxidation inhibition assay

Thirty microliters of different concentrated essential oils were incubated with 500 µl gelatin solution (1 mg/ml PBS) and 500 µl malondialdehyde solution (1.0 mg/ml in PBS) in a microplate. The microplate was incubated at ambient temperature for one day. Light absorbance recorded at 245 nm. The reduction in light absorbance at 245 nm was taken as the percentage inhibition in protein peroxide production as follows: percent inhibition = [(Absorbance in the absence of essential oil − Absorbance in the presence of essential oil)]/Absorbance in the absence of essential oil × 100. Butylated hydroxytoluene (BHT, 10 mg/ml) was used as control (Ansari et al., 2015).

### 2.7 Protein glycation inhibition assay

Thirty microliters of different concentrated essential oils were incubated with 500 µl gelatin solution (1.0 mg/ml PBS) and 500 µl glyceraldehyde solution (40 mg/ml in PBS). The microplate was then kept at ambient temperature for 24 hr. The solutions fluorescence was read at 370 nm (excitation) and 440 nm (emission). The percentage reduction in fluorescence intensity was taken as the percentage inhibition in protein glycation as follows: percent inhibition = [(fluorescence intensity in the absence of essential oil − fluorescence intensity in the presence of essential oil)]/fluorescence intensity in the absence of essential oil × 100. Aminoguanidine (10 mg/ml) was used as a positive control (Peng et al., 2008).

### 2.8 Amylase inhibition assay

Thirty microliters of different concentrated essential oils were added to 5.0 µl pancreatic amylase solution and incubated for 10 min at ambient temperature. The reaction catalyzed by amylase was immediately initiated by adding 20 µl starch solution (1.0 mg/ml) and incubated for 30 min at ambient temperature. The enzyme reaction was terminated by adding 20 µl HCl (1.0 M) to each well. Then, 100 µl of iodine reagent was added to each well. The absorbance of each solution in well was recorded at 580 nm, and the percentage of enzyme inhibition was observed as follows: percent inhibition − [(Absorbance in the absence of essential oil − Absorbance in the presence of essential oil)]/Absorbance in absence of the essential oil × 100. Acarbose (10 µg/ml) was used as control (Majeed et al., 2020).

### 2.9 Glucosidase inhibition assay

Thirty microliters of different concentrations of essential oils were added to 20 µl α‐glucosidase (50 µl/ml) in a microplate and then incubated for 15 min at ambient temperature. Then, 10 µl para‐nitrophenyl glucoside solution (10 mM) was added and incubated for 30 min at ambient temperature. Then, 30 µl Na_2_CO_3_ (100 mM) was added and the light absorbance at 405 nm was recorded. Acarbose (10 µg/ml) was added to some wells as control. The percent reduction in light absorbance at 405 nm was taken as the percent of enzyme inhibition as follows: percent inhibition = [(Absorbance in the absence of essential oil − Absorbance in the presence of essential oil)]/Absorbance in the absence of essential oil × 100 (Majeed et al., 2020).

### 2.10 Statistical analysis

All anti‐diabetic activity experiments were repeated three times. Statistical analysis of the treatments was performed by one‐way analysis of variance (ANOVA) using software SPSS software (SPSS Inc., Chicago, IL, USA), with *p* < .05 considered significant.

## 3 RESULTS AND DISCUSSION

### 3.1 Essential oil compositions

The main components of ODEO were carvacrol (40.38%), thymol (37.77%), gamma‐terpinene (12.72%), para‐cymene (5.045), sabinene (1.53%), and limonene (1.16%) (Table 1). The basic ingredients of TAEO were thymol (86.41%), para‐cymene (5.21%), gamma‐terpinene (3.64%), and carvacrol (3.14%). (Table 1). The main components of TKEO were carvacrol (43.39%), geraniol (14.55%), linalool (6.41%), caryophyllene (4.96%), citronellol (4.81%), alpha‐terpinene (4.76%), 4‐terpineol (4.63%), para‐cymene (3.38%), thymol methyl ether (2.62%), limonene (2.20%), borneol (1.49%), thymol (1.18%), alpha‐thujene (1.03%), beta‐pinene (1.03%), beta‐myrcene (1.03%), and isospathulenol (1.01%) (Table 1). The major constituents of ZMEO were carvacrol (52.365), thymol (14.95%), spathulenol (13.61%), caryophyllene oxide (4.37%), caryophyllene (3.29%), para‐cymene (3.24%), globulol (1.47%), aromadendrene epoxide (1.29%), thymol methyl ether (1.255), gamma‐terpinene (1.23%), and alpha‐terpinene (1.06%) (Table 1). Carvacrol was the main phytoconstituents of ODEO, TKEO, and ZMEO whereas thymol was the major constituent of TAEO as reported by others (Jamali et al., 2018; Kavoosi & Rabiei, 2015; Ranjbaran et al., 2019).

**TABLE 1:** Chemical composition (%) of essential oils from *Oliveria decumbens* (ODEO), *Trachyspermum ammi* (TAEO), *Thymus kotschyanus* (TKEO), and *Zataria multiflora* (ZMEO)

Compounds	Formula	RT	RI	ODEO	TAEO	TKEO	ZMEO
alpha‐Thujene	C_10_H_16_	3.44	924	0.00	0.00	1.03	0.00
alpha‐Pinene	C_10_H_16_	4.80	931	0.13	0.00	0.69	0.00
Sabinene	C_10_H_16_	5.85	958	1.53	0.00	0.00	0.00
beta‐Pinene	C_10_H_16_	6.19	975	0.21	0.21	1.03	0.00
beta‐Myrcene	C_10_H_16_	6.19	990	0.00	0.00	1.03	0.00
alpha‐Terpinene	C_10_H_16_	6.94	1,015	0.22	0.00	4.76	1.06
para‐Cymene	C_10_H_14_	7.20	1,023	5.04	5.21	3.38	3.24
Limonene	C_10_H_16_	7.32	1,026	1.16	0.00	2.20	0.00
gamma‐Terpinene	C_10_H_16_	8.29	1,056	12.72	3.64	0.00	1.23
Linalool	C_10_H_18_O	9.80	1,098	0.00	0.00	6.41	0.00
Borneol	C_10_H_18_O	12.29	1,164	0.00	0.00	1.49	0.00
Terpinen‐4‐ol	C_10_H_18_O	12.75	1,175	0.00	0.00	4.63	0.00
Citronellol	C_10_H_20_O	8.61	1,189	0.00	0.00	4.81	0.00
Thymol methyl ether	C_11_H_16_O	15.46	1,232	0.00	0.00	2.62	1.25
Carvone	C_10_H_14_O	15.70	1,243	0.00	0.40	0.00	0.00
Geraniol	C_10_H_18_O	16.77	1,253	0.00	0.00	14.55	0.00
Thymol	C_10_H_14_O	17.85	1,292	37.77	86.41	1.18	14.95
Carvacrol	C_10_H_14_O	18.49	1,311	40.38	3.14	43.39	52.36
Spathulenol	C_15_H_24_O	20.25	1,352	0.00	0.00	0.00	13.61
Globulol	C_15_H_26_O	20.58	1,370	0.00	0.00	0.00	1.47
Caryophyllene	C_15_H_24_	22.64	1,417	0.00	0.00	4.96	3.29
Isospathulenol	C_15_H_24_O	25.85	1,437	0.00	0.00	1.01	0.92
Aromadendrene epoxide	C_15_H_24_O	28.27	1,506	0.00	0.00	0.00	1.29
Caryophyllene oxide	C_15_H_24_O	29.09	1,580	0.00	0.00	0.00	4.37
Total				99.34	99.55	99.76	99.80
Monoterpenes				21.01	9.05	14.12	5.53
Monoterpenoids				78.15	89.95	79.09	68.57
Sesquiterpenes				0.00	0.00	4.96	3.29
Sesquiterpenoids				0.00	0.00	1.01	21.67

Note

The essential oils were identified by the GC‐MS software by comparing the retention times and fragmentation patterns of the related peaks with those reported in the libraries of Wily and NIST.

### 3.2 Physico‐chemical properties of gelatin‐pectin‐essential oil solution

**FIGURE 1:**
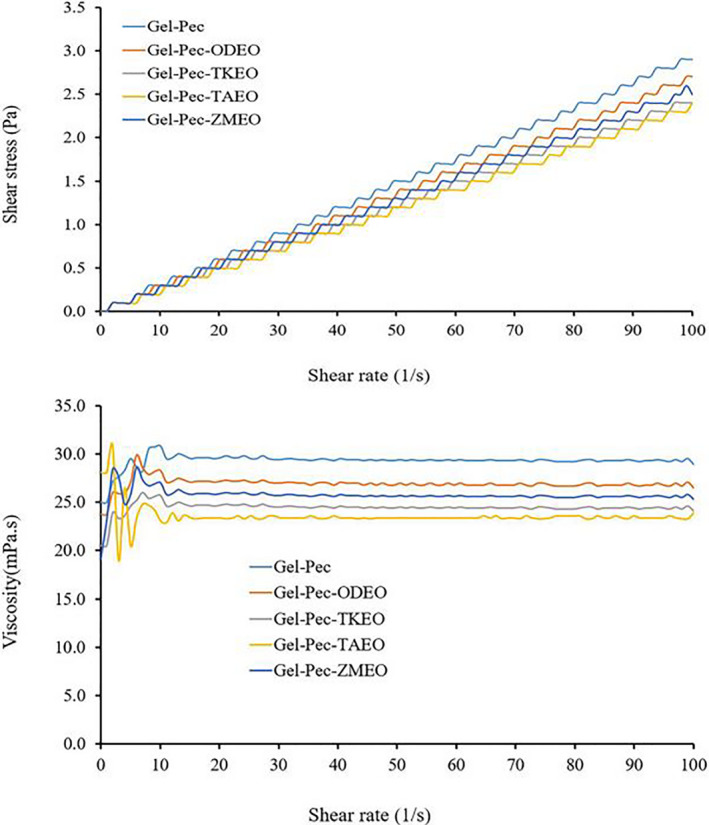
Shear stress and viscosity of the gelatin‐pectin solution and gelatin‐pectin solutions incorporated with essential oil from *Oliveria decumbens* (ODEO), *Trachyspermum ammi* (TAEO), *Thymus kotschyanus* (TKEO), and *Zataria multiflora* (ZMEO)

Gelatin‐pectin solutions displayed a Newtonian fluid nature with low conductivity, acidic pH, low surface tension, low zeta‐potential, and nanoscale particle sizes (Table 2). The addition of essential oils had no significant effects on conductivity, viscosity, surface tension, size and zeta‐potential, electrical net surface charge of the gelatin‐pectin particles in the solution. The rheological properties are key parameters for particle stability; hence, they were measured to determine the tendency of particles for aggregation or dispersion in biological fluids as well as their ability to deliver payloads. The bioactivity of essential oils may change with their physical properties. Different studies have reported higher bioactivity for essential oils at acidic pH. Because, at low pH, essential oils are more hydrophobic and will be passed through the cell membranes easily (Liu et al., 2020). Gelatin‐pectin solutions dissolved in acetic acid solution display low Newtonian behavior. In the Newtonian fluid, the viscosity of the solution is independent of the shear rate. The shear stress was increased by an increase in the shear rate in a linear manner. Thus, viscosity remained constant (Figure 1). The increase in the shear stress may be related to a negative charge and the small sizes of the particles and to the increased interfacial surface and finally causing a rise in the friction force between particles. By the increase in the shear rate, the contact between particles will be increased and the result is the increase in shear stress (Pitois & Rouyer, 2019).

**TABLE 2:** Properties of gelatin‐pectin composite solution incorporated with essential oil from *Oliveria decumbens* (ODEO), *Trachyspermum ammi* (TAEO), *Thymus kotschyanus* (TKEO), and *Zataria multiflora* (ZMEO)

Properties	Pure gelatin‐pectin	Gelatin‐pectin‐ODEO	Gelatin‐pectin‐TKEO	Gelatin‐pectin‐TAEO	Gelatin‐pectin‐ZMEO
pH	3.05 (0.3)^a^	2.40 (0.2)^a^	2.62 (0.1)^a^	2.51 (0.2)^a^	2.62 (0.1)^a^
Conductivity (µS/cm)	278 (10))^a^	272 (12)^a^	269 (9.0)^a^	265 (13)^a^	268 (8.0)^a^
Zeta‐potential (‐mV)	14.2 (1.6)^a^	15.5 (1.7)^a^	15.8 (1.4)^a^	16.5 (1.8)^a^	16.9 (2.0)^a^
Particle size (nm)	336 (12)^a^	315 (10)^a^	313 (7.0)^a^	317 (9.0)^a^	319 (11)^a^
Viscosity (mPa.s)	28.5 (3.7)^a^	26.2 (4.2)^a^	24.8 (3.8)^a^	23.7 (4.0)^a^	25.3 (4.5)^a^
Surface tension (mN/m)	23.5 (3.0)a	20.2 (2.7)^a^	19.7 (2.5)^a^	18.9 (2.6)^a^	19.6 (2.4)^a^

Note

The values are expressed as means (standard deviation) of three replicates. Mean values with different letters within a row are significantly different by Tukey test at (*p* < .05).

### 3.3 Physico‐chemical properties of gelatin‐pectin‐essential oil nanoparticles

**FIGURE 2:**
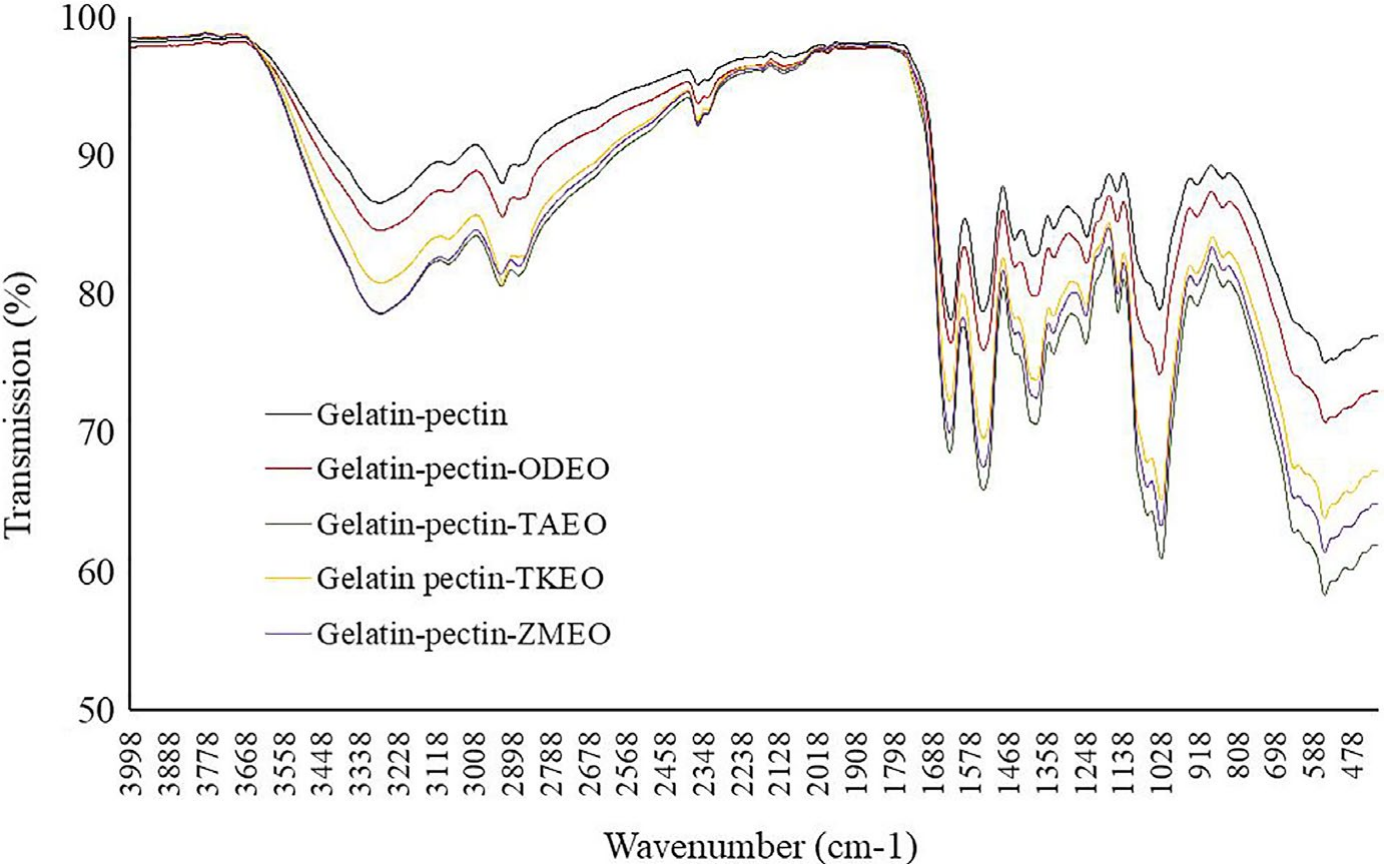
FTIR spectrum of gelatin‐pectin and gelatin‐pectin solutions incorporated with Oliveria (ODEO), Thymus (TKEO), Trachyspermum (TAEO), and Zataria (ZMEO) essential oil

The interaction among gelatin, pectin, and essential oils was analyzed by FTIR spectroscopy. The FTIR spectra of gelatin‐pectin and gelatin‐pectin‐essential oils to some extent are similar (Figure 2). The band at 3,600 to 3,300 cm^−1^ is related to water OH stretch or to phenol of ZMEO or to hydrogen bonding between dendrosome polymers. The bands from 3,000 to 2,800 cm^−1^ are attributed to CH stretch in CH, CH_2_, and CH_3_ of aliphatic groups. The stretch bands from 2,900 to 2,700 cm^−1^ are related to O‐CH_3_ in the methyl ester. The band at 2,400–2,200 cm^‐1^ is due to C = O and COO groups in the carbonyl and carboxyl functional groups. The band at the regions of 1,700 to 1,500 cm^−1^ is attributed to the asymmetric stretch of C = O in ester while bands in the region of 1,500 and 1,400 cm^−1^ are the symmetric stretch of C = O in esters. The CH bending vibration of CH_2_ in the terpenes can be seen at 1,450 cm^−1^ and CH bending vibration of CH_3_ can be seen at 1,340 cm^−1^. The C–O stretch in the phenol and monoterpenoid compounds is seen at 1,200 to 1,100 cm^−1^. The bands at the 1,100 to 1,000 cm^−1^ can be attributed to the ether bond of C‐O‐C in the monoterpenoids (Cebi et al., 2019).

**FIGURE 3:**
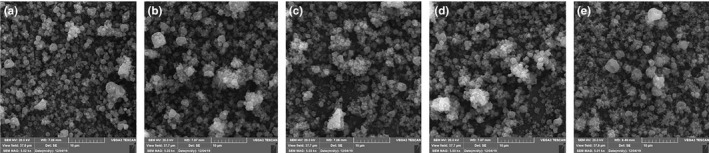
Scanning electron microscopy images of nanoparticles from gelatin ‐ pectin solution (a) and gelatin‐pectin solutions incorporated with Oliveria (b), Thymus (c), Trachyspermum (d), and Zataria (e) essential oil

*SEM* images of the electrospray‐gelatin‐pectin particles incorporated with the essential oils revealed that the particles had a size range of 300–600 nm and are globular in the shape (Figure 3). The particle sizes were higher than those estimated by LDS. This indicates that in electrospray and solvent evaporation, some particles have been attached to each other. The morphology of the nanoparticles has a significant impact on the uptake and transport of nanoparticles in the biological system (Baldino et al., 2019). Gelatin‐pectin‐essential oil nanoparticles have a spherical shape and are well dispersed. The globular shape of nanoparticles makes them the most capable of controlled release and protects the payloads because the spherical shape has the longest path to the movement of the entrapped extract in the nanoparticles and also the lowest contact area with the dispersed phase aqueous medium compared to other forms of nanoparticles (Loepfe et al., 2019). The extracted plants essential oils did not have a significant impact on the topology of the particles but improved their biological activity.

### 3.4 Glucose autoxidation inhibition

ODEO, TAEO, TKEO, and ZMEO at 110 to 130 µg/ml, inhibited glucose oxidation in the presence of copper ion in a similar manner, but at a lower level than EDTA (Table 3). Hydroxyl radicals can be generated from glucose autoxidation under the influence of transition metal ions like copper. The degree of benzoate hydroxylation reveals the extent of glucose oxidation. The decrease in benzoate hydroxylation in the presence of essential oils was mainly due to the scavenging of transition metals or hydroxyl radicals. Glucose autoxidation catalyzed by transition metals generates hydroxyl radicals and ketoaldehydes. Ketoaldehydes attach to proteins and lipids and produce an advanced glycation end product of proteins and lipids (Chetyrkin et al., 2011). Metal‐chelating agents inhibit glucose autoxidation and also reduce the covalent attachment of glucose (ketoaldehydes) to protein and lipids. As a result, the antioxidant essential oils by scavenging metal ions or hydroxyl radicals reduce glucose oxidation and protein glycation (Wu et al., 2011).

**TABLE 3:** Antioxidant and Antidiabetic activities (50% inhibitory concentration, IC_50,_ µg/ml) of essential oils from Oliveria *decumbens* (ODEO), *Trachyspermum ammi* (TAEO), *Thymus kotschyanus* (TKEO), and *Zataria multiflora* (ZMEO) incorporated in gelatin‐pectin solution

Diabetic marker	Control	Pure gelatin‐pectin	Gelatin‐pectin‐ODEO	Gelatin‐pectin‐TKEO	Gelatin‐pectin‐TAEO	Gelatin‐pectin‐ZMEO
Glucose oxidation	110 (6.0)^a^	755 (17)^c^	137 (5.6)^b^	143 (7.5)^b^	150 (7.0)^b^	130 (5.5)^b^
Lipid peroxidation	77 (4.2)^a^	780 (20)^c^	128 (6.7)^b^	123 (6.0)^b^	127 (6.5)^b^	124 (5.0)^b^
Protein peroxidation	146 (6.3)^a^	720 (23)^b^	150 (7.0)^a^	168 (7.5)^a^	160 (5.8)^a^	151 (5.5)^a^
Protein glycation	144 (5.0)^a^	694 (17)^c^	145 (5.6)^a^	169 (7.3)^b^	163 (8.0)^b^	165 (7.7)^b^
Amylase inhibition	126 (7.0)^a^	532 (15)^c^	223 (8.4)^b^	229 (9.0)^b^	218 (7.3)^b^	216 (7.7)^b^
Glucosidase inhibition	139 (6.5)^a^	512 (16)^c^	220 (7.2)^b^	238 (8.8)^b^	212 (9.0)^b^	219 (10)^b^

Note

The concentration of samples that provide 50% oxidant inhibition (IC_50_) was calculated from the graph that plotted the oxidant inhibition percentage against different concentrations of each extract. The values are expressed as means (standard deviation) of three replicates. Mean values with different letters within a row are significantly different by Tukey test at (*p* < .05).

### 3.5 Lipid peroxidation inhibition

ODEO, TAEO, TKEO, and ZMEO inhibited lipid peroxidation stimulated by copper ion at 120 to 130 µg/ml but at a lower level than positive control, BHT (Table 3). The accumulation of superoxide and hydroperoxide radicals under hyperglycemia leads to lipid peroxidation initiation and propagation, especially in conjugated polyunsaturated fatty acids. This process causes the production of reactive aldehydes such as glyoxal, methylglyoxal, and malondialdehyde that create lipoxidation products (Moldogazieva et al., 2019). Lipoxidation leads to an increase in protein aggregation that results in a dramatic change in cell signaling which causes cell damages and cell death. This process is associated with numerous pathological disorders like neurodegenerative diseases, atherosclerosis, inflammation, and vascular complications of diabetes (Tangvarasittichai, 2015). Medicinal plants and phytochemicals with strong antioxidant activity can be used as antioxidant therapy against lipid peroxidation (Foti & Ingold, 2003). The antioxidant ability of phytochemicals as polyphenols, flavonoids, and terpenoids is because of the redox potentials that make them act as electron donors, reducing power, proton donors, and singlet oxygen quenchers (Salehi et al., 2019). The strong antioxidant capacity of studied essential oils against lipid peroxidation has been related to phytochemical compounds like thymol and carvacrol or the synergism between para‐cymene and gamma‐terpinene.

### 3.6 Protein oxidation inhibition

ODEO, TAEO, TKEO, and ZMEO reduced protein oxidation induced by malondialdehyde at 140 to 170 µg/ml similar to positive control (Table 3). Diabetes is accompanied by hyperglycemia and superoxide and hydroperoxide production which can oxidize proteins, peptides, and amino acids like cysteine, methionine, tyrosine, histidine, and tryptophan. Protein oxidation and protein hydroperoxide is the covalent modification of amino acid side chains by superoxide and hydroperoxide. Oxidation of amino acids results in protein cross‐linkage and protein fragmentation (Davies, 2016). Oxidative modification of proteins might change protein properties, such as conformation, folding, stability, activity, and resistance to proteolysis that could be associated with various pathological consequences such as age‐related disease (Khyade, 2019). Phytochemicals with a high antioxidant capacities have improved the proteins oxidation stability (Cheng et al., 2020). Polyphenols present in phytochemicals inhibit protein aggregation and also breakdown the protein aggregates due to their antioxidant capacities (Debnath et al., 2016). The plants used in this study, had a high level of phenolic compounds and high antioxidant capacity and their capacities in protein oxidation inhibition may be related to their monoterpenoid contents.

### 3.7 Protein glycation inhibition

ODEO, TAEO, TKEO, and ZMEO diminished protein glycosylation in the presence of glyceraldehyde at 145–170 µg/ml similar to aminoguanidine (Table 3). Protein glycosylation is the reaction between reducing sugars (glucose, fructose, and glyceraldehyde) and amine groups of amino acid side chains leading to the formation of amadori products and over a long time produces glycosylation products (Justino et al., 2019). Glycosylation of proteins and enzymes causes the progression of pathological disorders known as atherosclerosis, diabetes, neurodegenerative and renal diseases. Protein glycosylation inhibitors with their strong antioxidant activities, scavenge free radicals and may break cross‐linkages between sugars and proteins and thus interfere with the attachments of reducing sugars with proteins by preventing amadori product formation (Younus & Anwar, 2016). Phytochemicals as nontoxic, low‐cost, and ingestible products like essential oils with broad antioxidant activity against reactive oxygen and nitrogen radicals by scavenging free radicals (Wu et al., 2009) and disrupting protein‐sugar interaction (Peng et al., 2008) have displayed antiglycation effects.

### 3.8 Amylase inhibition

ODEO, TAEO, TKEO, and ZMEO inhibited amylase activity at 210–230 µg/ml in a similar manner but at a lower levels as compared with acarbose (Table 3). Amylases catalyze the hydrolysis of alpha‐glycosidic linkages in starch and glycogen. Inhibition of amylase is considered a useful strategy for the treatment of carbohydrate metabolism disorders and related diseases like diabetes, obesity, and tooth decay (Sales et al., 2012). Among the phytochemicals that have been studied, phenolic compounds (Kwon, Apostolidis, & Shetty, 2008) and flavonoids (Majeed et al., 2020) have demonstrated the highest inhibitory effects against amylases. Phenolic compounds with noncompetitive or uncompetitive inhibition strategies prevent amylase activity (Franco et al., 2020).

### 3.9 Glucosidase inhibition

ODEO, TAEO, TKEO, and ZMEO inhibited glucosidase activity at 210–240 µg/ml similarly but at lower levels as compared to acarbose (Table 3). Glucosidase is the key enzyme catalyzing the breakdown of carbohydrates such as starch and disaccharides at the final step of digestive process. Glucosidase inhibitors can retard glucose release and absorption and as a result suppress postprandial hyperglycemia (Kumar et al., 2011). In order to search for foods for diabetic patients, various studies have been made to identify glucosidases from natural resources (Brown et al., 2017). Phytoconstituents, such as anthocyanin, glycoside, flavonoid, alkaloid, terpenoids, and phenolic compounds, have been suggested that effectively inhibit glucosidase (Rouzbehan et al., 2017). Results obtained from the present study show that plant essential oils possess bioactive components with antioxidant along with amylase and glucosidase inhibition properties. Therefore, these plants can be used as an important approach in controlling blood glucose in diabetes patients with no adverse effects.

## 4 CONCLUSION

In summary, thymol, carvacrol, gamma‐terpinene, para‐cymene, geraniol, and spathulenol are the main components of essential oils. All essential oils extracted from the plants used in this study, displayed strong antioxidant activity against glucose oxidation, lipid peroxidation, protein oxidation, protein glycation, and had anti‐diabetic effects against amylase and glucosidase activities. With these antioxidant and antidiabetic activities, the incorporation of the thymol and carvacrol bearing essential oils in the gelatin‐pectin composite provides a functional biomaterial for food product stability food encapsulation, wound healing, and cosmetics materials. However, more research is needed to be carried out on the intracellular antidiabetic activity of these essential oils to examine the molecular mechanisms of their antidiabetic activities at the cellular level or in living organisms. In addition, the efficacy of these composites in the treatment of diabetic foot ulcers should be investigated. Furthermore, the antidiabetic activity of pure chemical compounds and the synergistic effects among these compounds must be taken into account.

## CONFLICTS OF INTEREST

The authors confirm that they have no conflicts of interest.

## AUTHOR CONTRIBUTIONS

GK and RS conceived and designed research and conducted experiments and provide reagents and analytical tools. GK and RSh participated in writing the manuscript and its revisions. Research data are not shared.

## Supporting information

Figures S1–S4
