# Supplementary material for: In vitro antioxidant and antidiabetic activity of essential oils encapsulated in gelatin‐pectin particles against sugar, lipid and protein oxidation and amylase and glucosidase activity
Source: Food Sci Nutr. 2020 Oct 7;8(12):6457–66. doi: 10.1002/fsn3.1935 (PMC7723207; doi:10.1002/fsn3.1935)
Supplement: Supplementary file 1 — Figures S1–S4 [file FSN3-8-6457-s001.doc]

**In vitro antioxidant and anti-diabetic activity of essential oils encapsulated in gelatin-pectin particles against sugar, lipid and protein oxidation and amylase and glucosidase activity**

Roghayeh Siahbalaei^1^, Gholamreza Kavoosi^1*^, Raheleh Shakeri^2^

1. Institute of Biotechnology, Shiraz University, Shiraz, 71441-65186, Islamic Republic of Iran

2. Department of Biological Sciences and Biotechnology, Faculty of Sciences, University of Kurdistan, Sanandaj, Iran

*Corresponding author: [ghkavoosi@shirazu.ac.ir](mailto:ghkavoosi@shirazu.ac.ir)

**Abstract**

The in vitro antioxidant and anti-diabetic activities of *Oliveria decumbens*, *Thymus kotschyanus*, *Trachyspermum ammi*, and *Zataria multiflora* essential oils incorporated into gelatin-pectin composite were investigated. The gas-chromatography-mass spectrometry characterization revealed that thymol (1.2-86.4%), carvacrol (3.2-52.4%), gamma-terpinene (0.0-12.7%), para-cymene (3.2-5.2%), geraniol (0.0-14.5%), and spathulenol (0.0-13.6%) are the major constituents of the essential oils. Gelatin-pectin composite incorporated with the essential oils exhibited acidic pH (2.40-3.04), low conductivity (265-278 µS/cm), low surface tension (19.0-23.5 mN/m), low Newtonian viscosity (23.7-28.5 mPa.s), negative zeta-potential (14.2 -16.9 mV), and nanoscale particle size (313-336 nm). These rheological properties result in the production of globular gelatin-pectin nanoparticles with a size range of 500-700 nm. The FTIR spectra of gelatin-pectin and gelatin-pectin-essential oils to some extent were similar, suggesting the non-covalent interactions between them. Gelatin-pectin composite incorporated with the essential oils displayed anti-glucose oxidation (130-150 µg/mL) anti-lipid peroxidation (120-130 µg/mL), anti-protein oxidation (150-168 µg/mL), and anti-protein glycation (145-170 µg/mL) as well as anti-amylase (216-230 µg/mL), and anti-glucosidase (212-238 µg/mL) activity. The essential oils strongly improved the antioxidant capacity of the gelatin-pectin composite so strongly which can be recommended as natural compound for oxidative stress management.

**Keywords**: Essential oils; Gelatin; Pectin; Glucose oxidation; Lipid oxidation; Protein oxidation

|  |
| --- |
| Fig.S1. Total ion chromatogram (TIC) of Oliveria documbense essential oil anlayzed by gas chromatigraphy-mass spectrometry (GC-MS). |

|  |
| --- |
| Fig.S2. Total ion chromatogram (TIC) of Trachyspermum ammi essential oil anlayzed by gas chromatigraphy-mass spectrometry (GC-MS). |

|  |
| --- |
| Fig.S3. Total ion chromatogram (TIC) of Thymus kotschyanus essential oil anlayzed by gas chromatigraphy-mass spectrometry (GC-MS). |

|  |
| --- |
| Fig.S4. Total ion chromatogram (TIC) of Zataria multiflora essential oil anlayzed by gas chromatigraphy-mass spectrometry (GC-MS). |
